# Longitudinal alterations in brain networks and thalamocortical connectivity in paediatric focal epilepsy: a structural connectomics pilot study

**DOI:** 10.1093/braincomms/fcaf081

**Published:** 2025-02-27

**Authors:** Aswin Chari, Rory J Piper, Rachel Wilson-Jeffers, Michelle Ruiz-Perez, Kiran Seunarine, M Zubair Tahir, Chris A Clark, Richard Rosch, Rod C Scott, Torsten Baldeweg, Martin M Tisdall

**Affiliations:** Developmental Neurosciences, Great Ormond Street Institute of Child Health, University College London, London WC1N 1EH, UK; Department of Neurosurgery, Great Ormond Street Hospital, London WC1N 3JH, UK; Developmental Neurosciences, Great Ormond Street Institute of Child Health, University College London, London WC1N 1EH, UK; Department of Neurosurgery, Great Ormond Street Hospital, London WC1N 3JH, UK; Developmental Neurosciences, Great Ormond Street Institute of Child Health, University College London, London WC1N 1EH, UK; Developmental Neurosciences, Great Ormond Street Institute of Child Health, University College London, London WC1N 1EH, UK; Developmental Imaging and Biophysics Unit, Great Ormond Street Institute of Child Health, University College London, London WC1N 1EH, UK; Developmental Neurosciences, Great Ormond Street Institute of Child Health, University College London, London WC1N 1EH, UK; Department of Neurosurgery, Great Ormond Street Hospital, London WC1N 3JH, UK; Developmental Imaging and Biophysics Unit, Great Ormond Street Institute of Child Health, University College London, London WC1N 1EH, UK; Department for Basic and Clinical Neuroscience, Institute of Psychiatry, Psychology and Neuroscience, King’s College London, London SE5 8AB, UK; Division of Neurology, Nemours Children’s Hospital, Wilmington, DE 19803, USA; Developmental Neurosciences, Great Ormond Street Institute of Child Health, University College London, London WC1N 1EH, UK; Developmental Neurosciences, Great Ormond Street Institute of Child Health, University College London, London WC1N 1EH, UK; Department of Neurosurgery, Great Ormond Street Hospital, London WC1N 3JH, UK

**Keywords:** focal epilepsy, epilepsy surgery, diffusion MRI, connectomics, paediatric epilepsy

## Abstract

Epilepsy is an archetypal brain network disorder characterized by recurrent seizures and associated psychological, cognitive and behavioural sequelae. Progressive brain network dysfunction may contribute to poorer outcomes following treatment, but this has never been tested in humans. In this structural connectomics pilot study, we assess whether there is progressive brain network dysfunction in a cohort of 23 children undergoing repeated multi-shell diffusion tensor imaging as part of their pre-surgical evaluation of focal epilepsy prior to epilepsy surgery. We analyse global and nodal graph metrics and thalamocortical connectivity, comparing the longitudinal changes to a cross-sectional cohort of 57 healthy controls. We identify no robust longitudinal changes in global or nodal network properties over a median of 1.15 years between scans. We also do not identify robust longitudinal changes in thalamic connectivity between scans. On sensitivity analyses, we identify increases in weighted degree at higher scales of brain parcellation and a decrease in the proportion of nodes with a low participation coefficient, suggesting progressive increases in intermodular connections. These findings of no or subtle structural longitudinal brain network changes over a relatively short timeframe indicate that either there are no progressive structural brain network changes over time in epilepsy or the changes appear over longer timescales. Larger studies with longer timeframes between scans may help clarify these findings.

## Teaser

In a cohort of 23 patients with epilepsy undergoing repeat diffusion MRI (1.15 years apart), Chari *et al.* build structural connectivity matrices. They identify no changes in graph metrics and thalamocortical connectivity between scans. This may indicate a stability of network measures over time or that these changes occur over a longer timeframe.

## Graphical Abstract

**graphical abstract:**
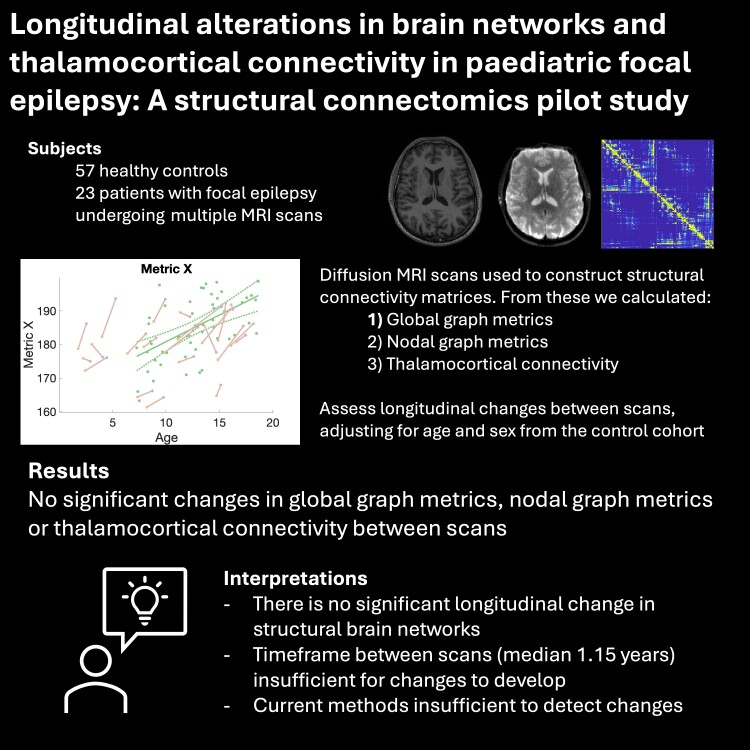

## Introduction

Epilepsy is the most common neurological condition in childhood, with a cumulative incidence of 0.66% at 10 years of age.^1^ Childhood epilepsy has long been associated with adverse neurodevelopmental outcomes,^2^ impacting functional, psychological and social outcomes in adulthood.^3^

Brain network dysfunction, a deviation from the normal developmental trajectories, may underlie both the propensity to seizures and the cognitive, psychological and social outcomes in epilepsy.^4-6^ There is evidence that brain network dysfunction exists at disease onset^7-9^ and an animal model has shown that ongoing seizures modify functional activities in brain regions outside of an epileptogenic focus.^10^ There is currently no direct human evidence of whether brain network dysfunction increases as a function of the duration of epilepsy,^11^ which may be attributable to a number of factors, including ongoing seizures and side effects of antiseizure medication (ASM). So far, human studies have identified patterns of cortical atrophy that may be downstream effects of network dysfunction.^12^

Progressive network dysfunction is important as it may contribute to worse long-term outcomes. There is evidence of progressive brain network dysfunction in individuals with Alzheimer’s disease, with the degree of decline being predictive of dementia severity.^13^ Postoperative seizure outcomes may be related to the severity of brain network dysfunction in focal epilepsy treated with surgical resection,^14-16^ which is supported by multiple studies demonstrating an inverse correlation between the duration of epilepsy and postoperative seizure freedom.^17-19^ Limiting brain network dysfunction may also be beneficial for other outcomes, such as developmental and cognitive outcomes.^6^ Therefore, understanding whether there is progressive network change has important therapeutic implications, including the consideration of whether surgical intervention to achieve seizure freedom should be considered prior to the establishment of drug resistance, which can often take many years to ascertain.^11^ There is also growing evidence that thalamocortical connectivity may be implicated in epilepsy and thalamic structural connectivity, and network properties have been shown to be deranged in focal epilepsies.^9,14^

This study examined longitudinal structural brain networks in children with drug-resistant focal epilepsy and sought to assess whether brain network properties and thalamic connectivity changed over time in individuals undergoing repeat diffusion MRI (dMRI) imaging as part of their pre-surgical evaluation for epilepsy surgery. We hypothesize that global network properties, nodal network properties and thalamocortical connectivity will progressively deviate from healthy controls over time, indicating that ongoing uncontrolled epilepsy, exposure to recurrent seizures and ASM contribute to progressive brain network dysfunction.

## Materials and methods

**Figure 1:**
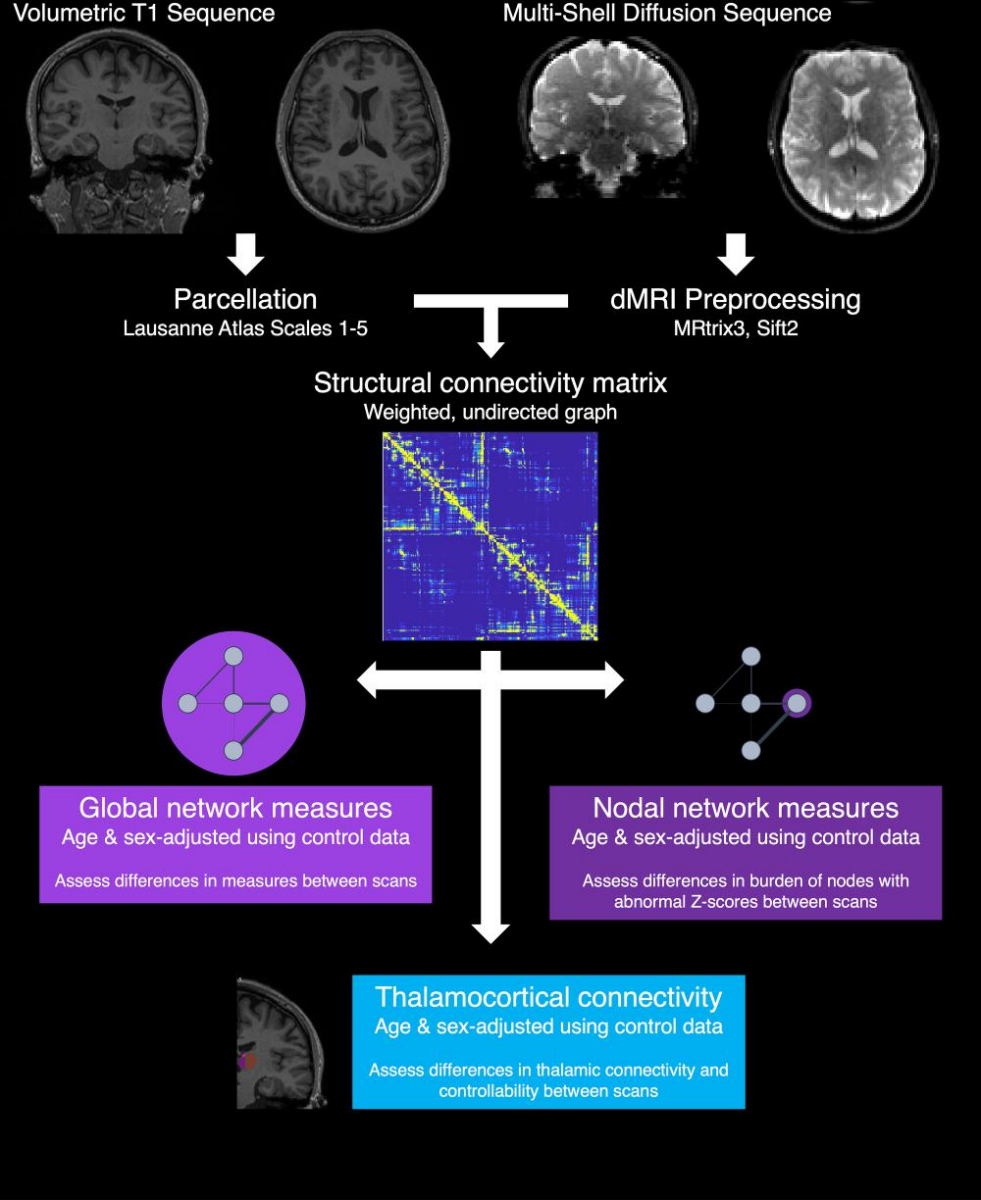
**Methods summary.** Structural and dMRI for healthy controls and patients (early and late scans) were pre-processed and co-registered using in-house scripts. MRTrix3 was then used to create structural connectivity matrices detailing the number of streamlines between each atlas parcel at each scale of the Lausanne atlas. These matrices were used to calculate global network measures, nodal network measures and thalamocortical connectivity, adjusted for age and sex using the control data set. Changes in the global metrics, burden of abnormal nodes in the nodal metrics and thalamocortical connectivity were compared between scans in the patient cohort to assess for longitudinal change.

This project received approval from the UCL Great Ormond Street Institute of Child Health Research and Development Department (Project ID 23NP01). This was a retrospective cohort study and is reported according to the STROBE guidelines (see checklist in Supplementary material). As we used routinely collected clinical data, the need for individual patient consent was waived. A summary of the methods is presented in Fig. 1 and detailed below.

### Patients

All patients undergoing resective epilepsy surgery for drug-resistant epilepsy at a single paediatric epilepsy surgery centre between 2015 and 2022 were eligible for inclusion. Patients were included if they underwent more than two diffusion MRI scans as part of their pre-surgical evaluation. If more than two data sets were available, we used the earliest and latest data sets.

### Controls

Controls were healthy children scanned as part of prior approved studies on the same scanner. These children did not have epilepsy or other neurological conditions. Some were siblings of children with sickle cell disease but did not have sickle cell disease themselves.

### MRI scanning

All patients and controls were scanned on the same Siemens Magnetom Prisma 3T MRI scanner with the same multi-shell multi-tissue dMRI protocol. The protocol included a T1 MPRAGE sequence and a multi-shell diffusion sequence employing a diffusion-weighted spin-echo single-shot 2D EPI acquisition, with multi-band radio frequency pulses to accelerate volume coverage along the slice direction. A multi-band factor of 2 was used to image 66 slices of 2 mm thickness with a 0.2-mm slice gap. Diffusion gradients were applied over two ‘shells’: *b* = 1000 and 2200 s/mm^2^, with 60 non-collinear diffusion directions per shell in addition to 13 interleaved *b* = 0 (non-diffusion-weighted) images. Other imaging parameters were: TR = 3050 ms, TE = 60 ms, field of view = 220 mm × 220 mm, matrix size = 110 × 110, in-plane voxel resolution = 2.0 mm × 2.0 mm, GRAPPA factor 2, phase-encoding partial Fourier = 6/8. An additional *b* = 0 scan was acquired, with an identical readout to the diffusion-weighted scan, but with the phase encode direction flipped by 180° (in the anterior-posterior direction), for correction of susceptibility-related artefacts.

### MRI processing

T1 MPRAGE images were processed using bias field correction and denoising tools from ANTS. Parcellation of the brain was performed using Connectome Mapper 3 (Tourbier 2022), which involves the use of Freesurfer (v 7.1.1) and conversion of parcels to the Lausanne scheme (2018 version)—including seven thalamic subregions^20^ on each side, the hippocampus divided into the head, body and tail,^21^ and four brainstem parcels.^22^ The spatial resolution of the atlas increased across five scales of the Lausanne parcellation scheme.

dMRI processing used a typical MRTrix3 process that involved denoising of dMRI (‘dwidenoise’), correction for inhomogeneity distortion (‘dwifslpreproc’) and bias correction (‘dwibiascorrect’). Whole-brain probabilistic tractography was performed by selecting 5 million tracts using 5ttgen, tckgen and tksift2 on MRTrix3.^23,24^ Rigid co-registration of the preoperative T1 MPRAGE and region-of-interest images was performed to move the structural parcellation into the dMRI space. Structural dMRI-derived connectomes at each Lausanne scale were constructed using the ‘tck2connectome’ command in MRTrix3.

### Analyses

Connectomes Scale 3 were used for the primary analysis as they represent a balance between large parcels that may improve signal-to-noise ratio and small parcels that may be more clinically relevant and surgically applicable. Graph and controllability measures (detailed below) were calculated using the brain connectivity toolbox^25^ and in-house scripts, respectively. These were chosen based on a combination of informed consideration of which measures we thought would change and prior literature.^14,26,27^

### Global metrics

In the first analysis, we calculated global network measures from each connectome (patients and controls), including:

- Mean degree: average weight of connections throughout the network.
- Transitivity: defined as ratio of triangles to triplets and is a measure of the clustering of the network.
- Modularity: the degree to which the network may be divided into subnetworks.
- Global efficiency: average inverse path length in the network.
- Mean average controllability: the ability of the brain to reach nearby, easy-to-reach neurophysiological states.
- Mean modal controllability: the ability of the brain to reach distant difficult-to reach neurophysiological states.

We then used generalized linear models (GLMs) to model the impact of age and sex on these network measures in the control data set and applied these model parameters to the patients at both scan time points to calculate age- and sex-adjusted residuals. We then compared the distribution of these residuals between early and late scans. We tested for statistical differences using a non-parametric paired Wilcoxon signed rank test and, to correct for multiple comparisons, used a threshold of *P* < 0.01 to denote significance. We estimated effect size using Cohen’s *d*.

We assessed whether the differences in residuals varied by subgroups (histopathology and seizure freedom) using Kruskal–Wallis test. Again, to correct for multiple comparisons, a threshold of *P* < 0.01 was used to denote significance.

### Nodal metrics

In the second analysis, we calculated nodal network measures from each connectome (patients and controls), including:

- Degree: total number of streamlines connected to the node.
- Participation coefficient: a measure of the diversity of intermodular connections of individual nodes.
- Eigenvector centrality: a self-referential measure of centrality in which nodes have high eigenvector centrality if they connect to other nodes that have high eigenvector centrality.
- Local efficiency: global efficiency calculated on local neighbourhoods.
- Average controllability: the ability of the region to drive the brain to nearby easy-to-reach neurophysiological states.
- Modal controllability: the ability of the region to drive the brain to distant difficult-to-reach neurophysiological states.

Based on the work of Sinha *et al.*^15^, we sought to assess the burden of ‘abnormal nodes’ that fell outside a certain *Z*-score threshold, chosen at ±2.^15^ We again did this by calculating age- and sex-adjusted residuals for each network measure at each node using GLMs created from the controls. We then *Z*-scored the residuals in the controls and applied the mean and standard deviation to calculate *Z*-scores for each node in each patient and assessed the total number of nodes that fell outside the *Z*-score threshold (±2) in the controls and in the patients at early and late time points. We then compared the burden of nodes outside this threshold between scans using the paired non-parametric paired Wilcoxon signed rank test. To correct for multiple comparisons, we used a threshold of *P* < 0.01 to denote significance. We estimated effect size using Cohen’s *d*. To probe this further, we also assessed whether there were specific changes in the burden of nodes with *Z*-scores of >+2 or <−2.

### Sensitivity analyses

We conducted two sensitivity analyses. To assess the spatial specificity of the results, we repeated the analyses at the other scales of the Lausanne atlas (Scales 1, 2, 4 and 5). In addition, we noted that there was a pool of five patients who were significantly younger than our control data set; we therefore performed a second sensitivity analysis excluding these patients.

### Thalamic connectivity

Based on existing literature, we identified the thalamus and thalamocortical connectivity as a potentially important marker of progressive network dysfunction.^14,28,29^ To assess the change in thalamocortical connectivity between scans, we calculated *Z*-scores from age- and sex-adjusted residuals of the connectivity from the thalamus to each ipsilateral lobe using the healthy controls as a comparator. We assessed the change in *Z*-scores between scans. In addition, we assessed whether there were specific unique changes to thalamocortical connectivity between the thalamus and lobe affected by the epilepsy (i.e. the location of the resection) between scans by assessing whether the *Z*-score change of that lobe was different to the *Z*-score change from the other lobes within each patient.

Our previous work had identified changes in thalamic modal controllability between healthy controls and children with drug-resistant epilepsy. We finally sought to assess whether thalamic modal controllability changed between scans by assessing the change in age and sex-adjusted *Z*-score of the modal controllability of thalamic parcels between scans.

For both thalamic analyses, *Z*-scores between early and late scans were compared using a paired Wilcoxon signed rank test and, to correct for multiple comparisons, a threshold of *P* < 0.01 was used to denote significance. To correct for multiple comparisons, we used a threshold of *P* < 0.01 to denote significance. We estimated effect size using Cohen’s *d*.

### Statistical analyses

The statistical analyses conducted in each section have been outlined in the previous sections.

## Results

A total of 23 patients with drug-resistant epilepsy and 57 healthy controls were included in the study. Age ranges of patients and controls were different (Supplementary Fig. 1), and the interval between scans for patients was a median of 1.15 years (interquartile range 0.78–1.93 years). Full demographics, including postoperative seizure freedom and histological diagnosis, are provided (Supplementary Table 1).

### Global network measures reveal no changes in network properties between scans

**Figure 2:**
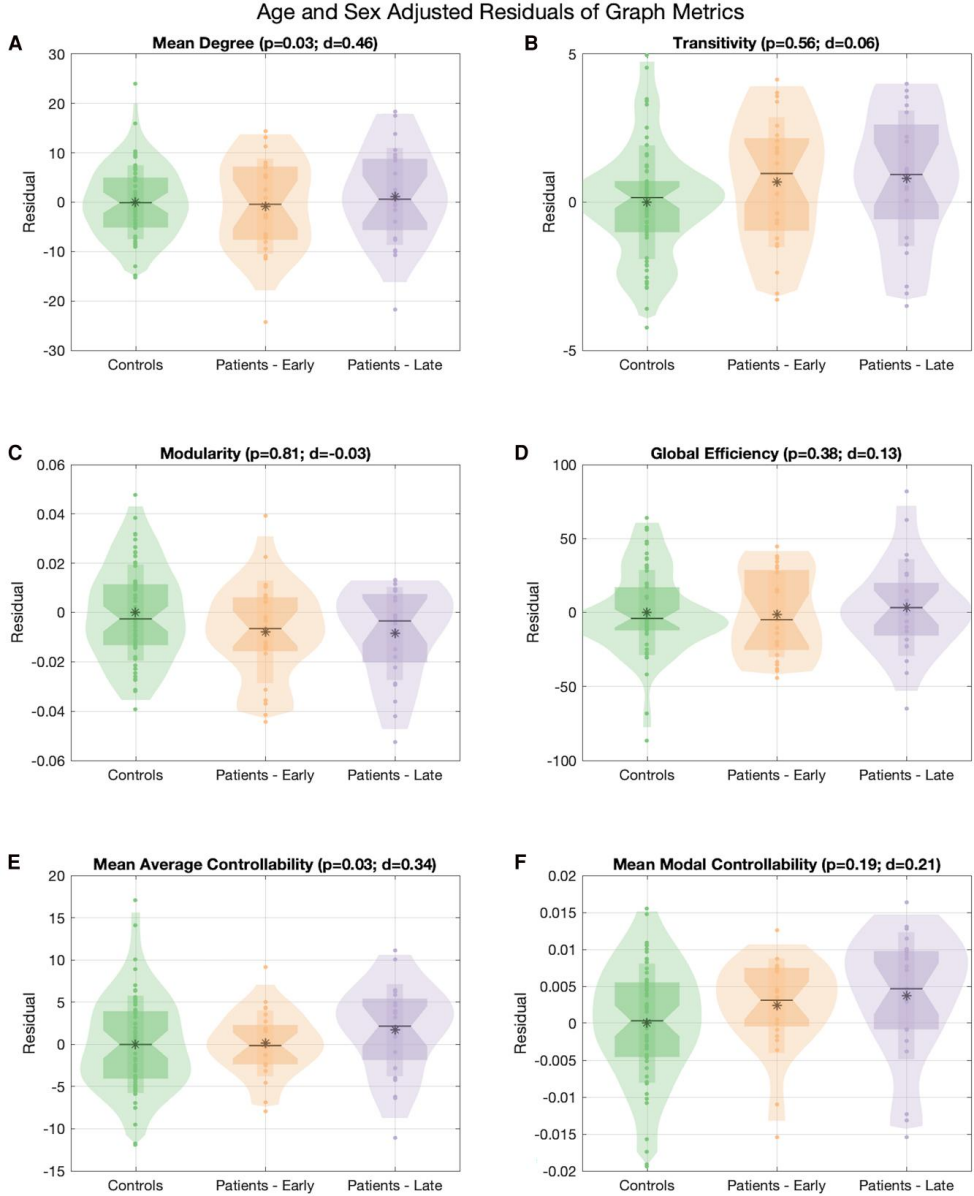
**Age- and sex-adjusted residuals of global metrics shown in the controls (*n* = 57) and patients (early and late scans, *n* = 23).** Residuals between early and late scans for (**A**) mean degree, (**B**) transitivity, (**C**) modularity, (**D**) global efficiency, (**E**) mean average controllability and (**F**) mean modal controllability were compared using a paired Wilcoxon signed rank test and, to correct for multiple comparisons, a threshold of *P* < 0.01 was used to denote significance. Statistical testing reveals no significant differences between early and late scans.

When assessing for global differences, there were no statistically significant changes in global network measures between scans (Fig. 2). There were notable increases in mean degree (*P* = 0.03, *d* = 0.46) and mean average controllability (*P* = 0.03, *d* = 0.34), both of which did not meet the predetermined thresholds for statistical significance.

When we modelled whether the change in residuals varied by histopathology and seizure freedom, again, none of the global metrics met our predetermined thresholds for statistical significance (Supplementary Fig. 2). However, it is interesting to note that there was a non-significant increase in the residual change for mean degree in the non-seizure-free cohort versus the seizure free (medians of 5.17 versus 0.82, *P* = 0.02).

### Nodal network measures reveal no change in the burden of abnormal nodes between scans

**Figure 3:**
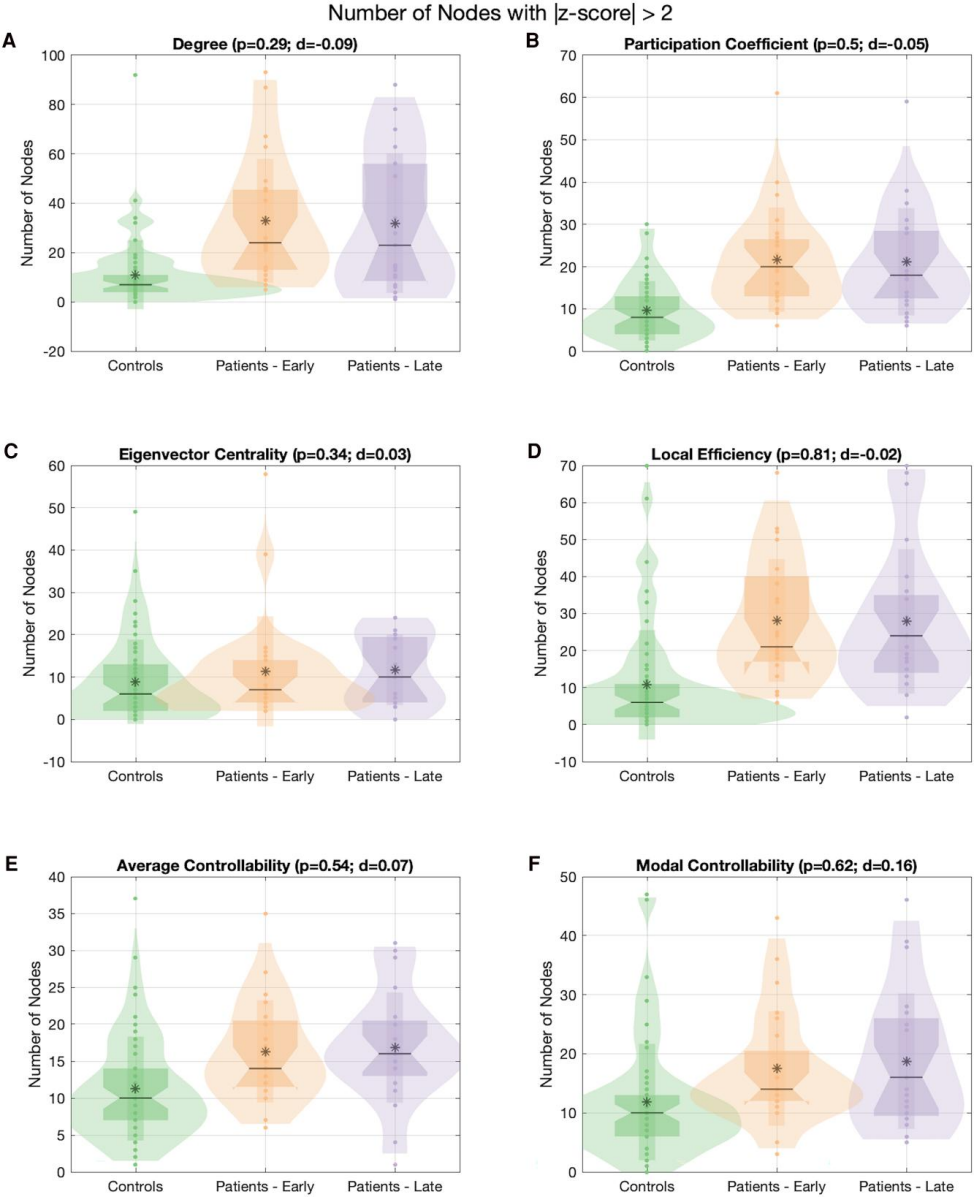
**Burden of abnormal nodes with |*Z*-score| > 2 shown in the controls (*n* = 57) and patients (early and late scans, *n* = 23).** The number of abnormal nodes between early and late scans for (**A**) degree, (**B**) participation coefficient, (**C**) eigenvector centrality, (**D**) local efficiency, (**E**) average controllability and (**F**) modal controllability were compared using a paired Wilcoxon signed rank test and, to correct for multiple comparisons, a threshold of *P* < 0.01 was used to denote significance. Statistical testing reveals no significant differences between early and late scans.

Between scans, there was no change in the burden of abnormal nodes across all the nodal measures tested (Fig. 3). We also looked at whether there was a change in the nodes specifically with *Z*-scores >2 or <−2. In this analysis, we identified a statistically significant decrease in the burden of participation coefficient nodes with a *Z*-score of < −2 (*P* = 0.0001; Supplementary Fig. 3).

### Sensitivity analyses reveal robust findings across scales of the Lausanne atlas

**Figure 4:**
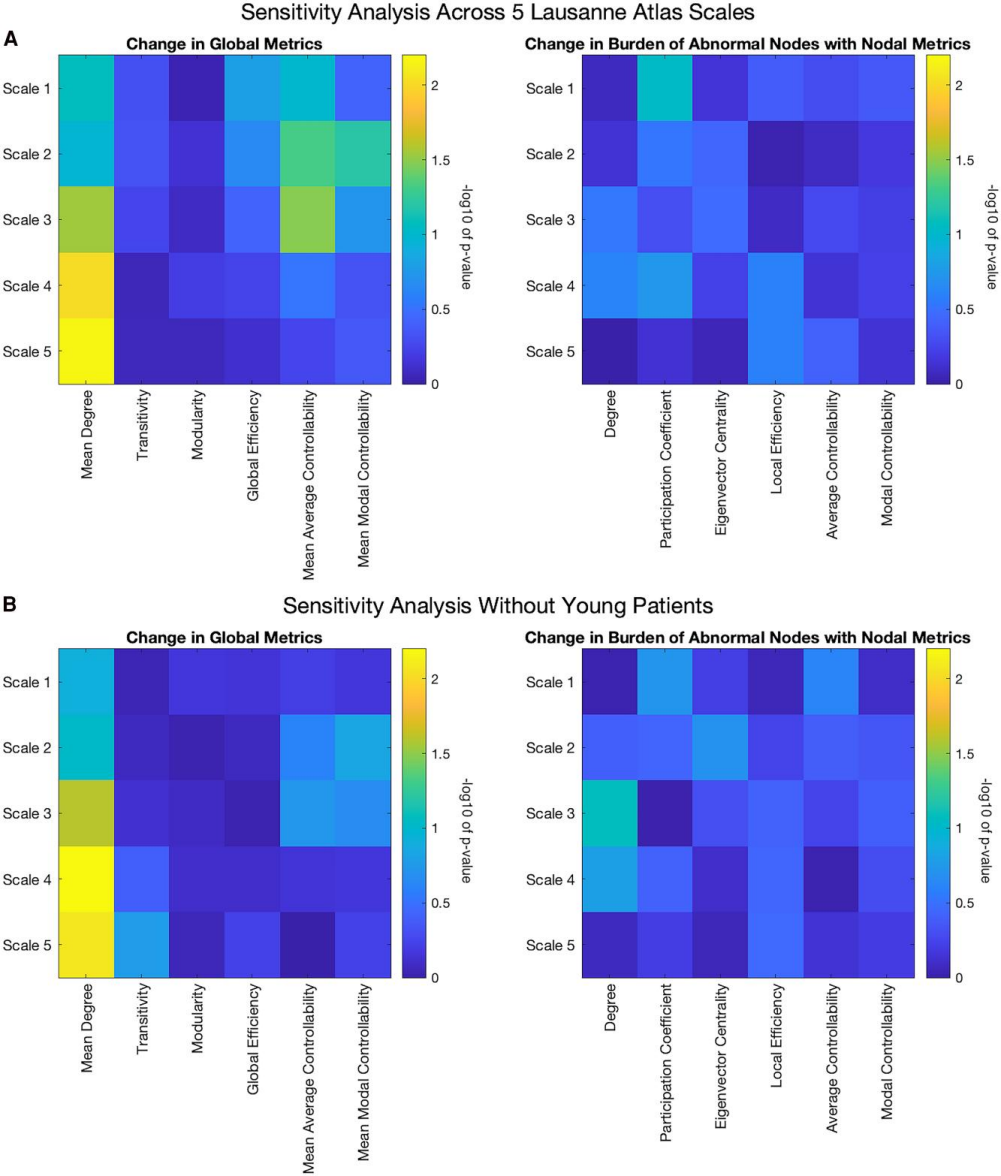
**Sensitivity analyses.** (**A**) Sensitivity analyses across all five scales of the Lausanne atlas revealing relatively consistent changes in global and nodal metrics across scales. This analysis includes all 23 patients. (**B**) Further consistency of findings when repeated, excluding the youngest patients for whom there was no comparative control data. This analysis excluded 5 patients and was therefore performed with 18 patients. The scales in all plots are −log_10_ of the *P*-value, with a value of 2 corresponding to a *P*-value of 0.01. Residuals between early and late scans were compared using a paired Wilcoxon signed rank test and, to correct for multiple comparisons, a threshold of *P* < 0.01 was used to denote significance. Across both sensitivity analyses, there were statistically significant increases in mean degree at Scales 4 and 5 of the Lausanne atlas (*P* < 0.01).

The first sensitivity analysis, conducted across Scales 1–5 of the Lausanne Atlas identified that some of the changes were consistent across spatial scales, whilst others were less so (Fig. 4A). At global level, mean degree was consistently increased between scans, especially at higher scales of the atlas; at Scales 4 and 5, these changes were statistically significant (*P* < 0.01). At the nodal level, there were no statistically significant changes in the burden of abnormal nodes across the scales. In the second sensitivity, their findings were consistent if the young patients (age <6, *n* = 5) were removed from the analysis (Fig. 4B).

### Thalamocortical connectivity and thalamic modal controllability are not significantly altered between scans

**Figure 5:**
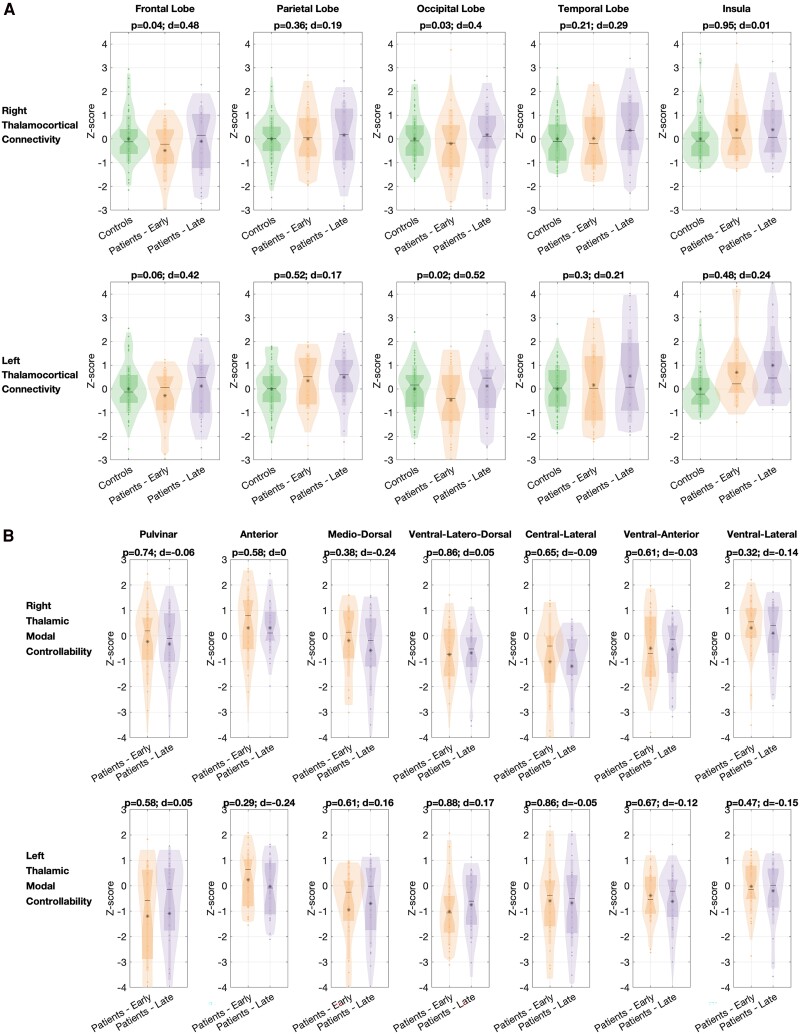
**Changes in thalamic connectivity and properties between scans.** (**A**) Changes in thalamic connectivity to ipsilateral lobes between scans in the patients (*n* = 23). No statistically significant changes were identified. (**B**) Changes in thalamic parcel modal controllability in patients (*n* = 23) are also not statistically significantly altered between scans. For both analyses, *Z*-scores between early and late scans were compared using a paired Wilcoxon signed rank test and, to correct for multiple comparisons, a threshold of *P* < 0.01 was used to denote significance.

On assessing the thalamocortical connectivity between each thalamus and its ipsilateral lobes, there were no statistically significant group-level differences in thalamocortical connectivity (Fig. 5A). Subtle differences in bilateral frontal-thalamic connectivity (*P* = 0.04, *d* = 0.48 for the right side and *P* = 0.06, *d* = 0.42 for the left side) and occipital-thalamic connectivity (*P* = 0.03, *d* = 0.40 for right side and *P* = 0.02, *d* = 0.52 for left side) were identified, all of which did not meet the predetermined criteria for statistical significance. None of these changes were graded (i.e. there was not a progressive increase between the controls, early patient and late patient scans), and therefore, it is not possible to interpret this as a progressive ‘abnormality’ of thalamocortical connectivity associated with disease course.

To assess whether there may be exaggerated connectivity changes isolated to the specific lobe to which the epilepsy was localized, we conducted a second within-patient normalization of *Z*-scores across the 10 lobes. The connectivity change in the affected lobes was no different from the other lobes (mean *z*-of-*z*-score 0.04, one-sample *t*-test *P* = 0.84), indicating that there were no focal longitudinal changes to thalamocortical connectivity to the lobe of seizure focus.

Given previous work outlining the importance of thalamic modal controllability,^14^ we finally assessed changes in thalamic modal controllability between scans. Again, there were no statistically significant changes in thalamic modal controllability *Z*-scores between scans (Fig. 5B).

## Discussion

### Is there longitudinal network dysfunction over time?

Numerous studies in the fields of functional MRI, dMRI, scalp EEG and intracranial EEG have established the network nature of epilepsy and the network dysfunction associated with various types of epilepsy.^6^ In this dMRI connectomics study, we aimed to assess longitudinal network property changes in children who have had multiple dMRI scans as part of their pre-surgical evaluation for drug-resistant focal epilepsy.

Although there seem to consistently be differences in the network properties between controls and patients (which were not systematically and statistically examined in this study but have been established in our prior work^14^), we did not demonstrate longitudinal changes in global network properties, burden of abnormal network nodes and thalamic connectivity between scans in the primary analyses in this study. However, we stress that this is in the context of a relatively short timeframe (median 1.15 years) between scans.

A key positive finding was the decrease in participation coefficient nodes with a *Z*-score < −2 (*P* = 0.0001, Supplementary Fig. 2). The participation coefficient measures how well a node is connected to other modules within the network, and therefore, this suggests an increased number of nodes with intermodular connections between scans, supporting the idea of a less well-organized network structure with progressive epilepsy. This was accompanied by a statistically not significant increase in the number of participation coefficient nodes with a *Z*-score >2 (*P* = 0.03, Supplementary Fig. 3).

In addition, we identified increases in mean degree at higher scales of the Lausanne atlas, and in our subgroup analysis, there was some indication that an increase in mean degree between scans was associated with a lack of seizure freedom (*P* = 0.02, Supplementary Fig. 2).

### Sensitivity analyses suggest increased degree between scans

The sensitivity analyses, conducted over other scales of the Lausanne atlas, revealed statistically significant increases in degree (number of streamlines) between scans at Scales 4 and 5 of the atlas (Fig. 4). Taken in context with our previous study demonstrating increased mean degree in children with drug-resistant focal epilepsy, this might reveal that there were changes that would, if they were to be given more time, result in network property changes.^14^

### Structural network dysfunction may develop over longer timescales and follow different trajectories

The underlying reasons for the lack of network property changes over such a short timeframe in the measures analysed in the primary analyses in this study could be manifold.

Firstly, it is possible that structural network changes do not worsen over time in focal epilepsy. However, the evidence of structural network changes^5,14,15^ and the clinical observations of worsening postoperative and neuropsychological outcomes with the duration of epilepsy^18,30^ in addition to the animal evidence^10^ point towards there being a strong possibility of progressive structural network dysfunction in epilepsy. It is likely that the structural network changes develop over a slower timescale than the median difference of 1.15 years between scans in our current cohort, and it would be powerful to have larger longitudinal cohorts with longer duration between scans to explore this further. Correlating the decline with changes in seizure and cognitive outcomes will shed insight into disease mechanisms and may guide treatment paradigms, including optimizing the timing of surgery to maximize both seizure freedom and neuropsychological outcomes.^11^

It is also possible that patients follow different trajectories that are not adequately captured by group-level analyses. Certainly, using large cross-sectional cohorts, Xiao *et al.*^12^ recently described three trajectories of brain region atrophy, that could be associate with differential structural network changes.^12^ Our current longitudinal cohort is too small to attempt to assess the network underpinnings of these trajectories.

An alternative explanation is that our methodology does not measure network changes. This could be at the level of the imaging modality, such that diffusion tensor imaging does not significantly change over time despite there being apparent functional network changes, or that the post-processing steps utilized, such as our choice to measure the number of streamlines rather than mean functional anisotropy that other groups have utilized.^31^ However, our previous work, using similar methodology, revealed robust differences between children with epilepsy and healthy controls, validating our methodological choices.^14^

### Limitations

Our study is limited by the small sample size and the convenience nature of the sample; all patients underwent multiple scans as part of their clinical pre-surgical evaluation, and it is therefore conceivable that there is a selection bias of more complex cases that necessitated multiple scans. Other reasons for multiple scans may include planned surveillance (e.g. of long-term epilepsy-associated tumours), requirement of additional sequences (e.g. for focal cortical dysplasia detection) or updating imaging for surgical planning. As alluded to above, the relatively short timeframe between scans is also a limiting factor in identifying structural network changes, which would be expected to develop over longer timeframes. It also remains to be firmly established whether any perceivable changes correlate with outcomes (e.g. postoperative seizure freedom).

Another key limitation is that we do not understand the test–retest variability of measuring structural brain networks and their properties; indeed, the variation we identified between scans could be a function of this variability rather than true change in the network and its properties between scans.

We also did not have longitudinal data from our controls, necessitating the use of a cross-sectional control cohort. However, we believe this paradigm has validity and allows us to model changes from the normal developmental trajectories in a cross-sectional cohort.^32^

## Conclusions

In this small pilot study, we demonstrate subtle, if any, longitudinal structural network changes in children with focal epilepsy undergoing multiple preoperative dMRI scans prior to epilepsy surgery. There is some evidence that ongoing epilepsy leads to increased weighted degree of structural brain networks (at certain scales of parcellation) and increases in the participation coefficient of nodes (more intermodular connections), which may underpin the network dysfunction contributing to poorer outcomes found with longer durations of epilepsy.^18,30^

## Supplementary Material

fcaf081_Supplementary_Data

## Data Availability

The code for MRI pre-processing and current analyses is available at https://github.com/aswinchari/LongitudinalNetworks. The structural connectivity matrices for the included patients across scales are available at https://osf.io/a28ex/. Raw MRI data are not provided due to patient confidentiality concerns.
