# Supplementary material for: Longitudinal alterations in brain networks and thalamocortical connectivity in paediatric focal epilepsy: a structural connectomics pilot study
Source: Brain Commun. 2025 Feb 27;7(1):fcaf081. doi: 10.1093/braincomms/fcaf081 (PMC11878571; doi:10.1093/braincomms/fcaf081)
Supplement: fcaf081_Supplementary_Data [file fcaf081_supplementary_data.docx]

**Longitudinal alterations in brain networks and thalamocortical connectivity in paediatric focal epilepsy: A structural connectomics pilot study**

**Supplementary Material**

**
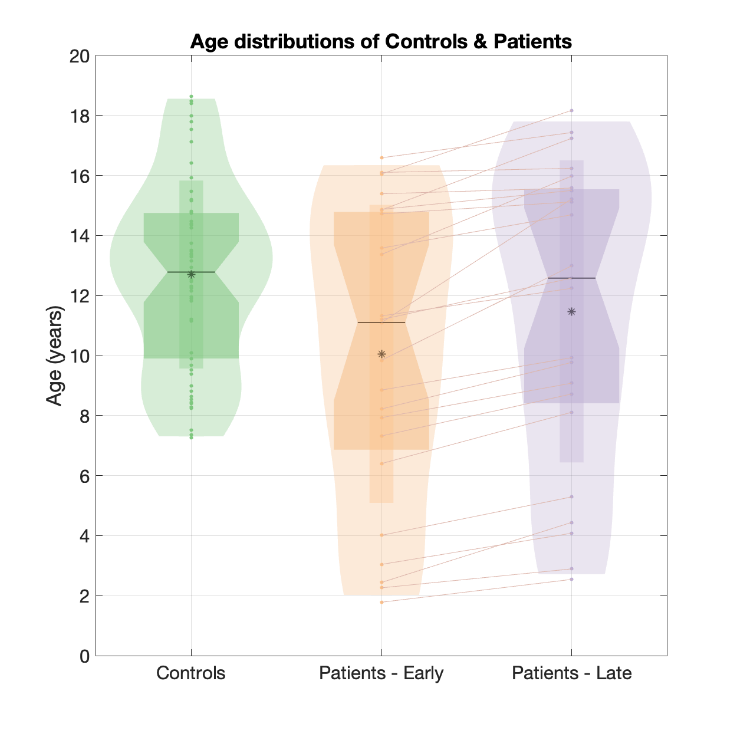
**

**Supplementary Figure 1:** Age ranges of the controls (n=57) and patients at early and late scan timepoints (n=23). The interval between scans for patients was a median of 1.15 years (IQR 0.78 – 1.93 years).


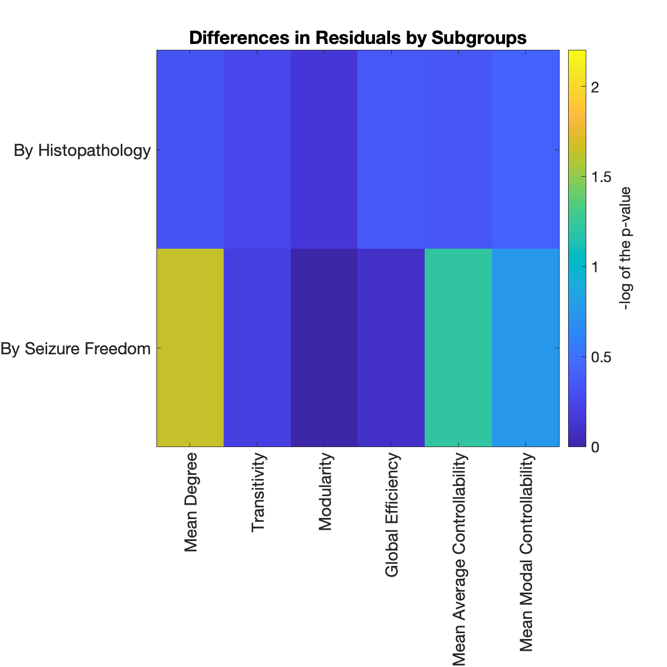


**Supplementary Figure 2:** Subgroup analysis to assess if the differences in residuals of the global metrics were different by histopathology or seizure freedom status using. Statistical analysis was performed using the Kruskal Wallis test. The scale is -log_10_ of the p-value, with a value of 2 corresponding to a p-value of 0.01. Although none of the analyses were statistically significant, there was an indication that there was an increase in the residuals for mean degree between scans in the non-seizure free cohort compared to the seizure free cohort (medians of 5.17 vs 0.82, p = 0.02).


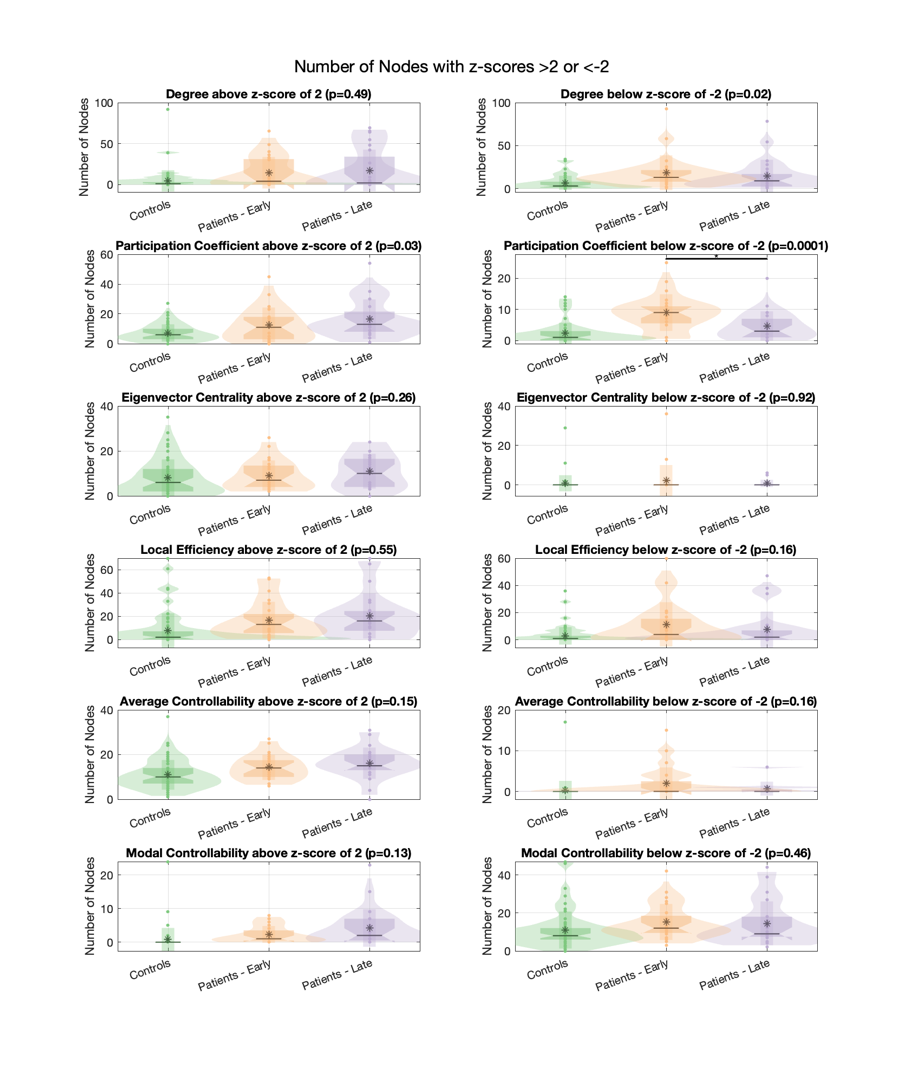


**Supplementary Figure 3: Burden of abnormal nodes with z-score > 2 or <-2.** Statistical analysis was performed using a paired Wilcoxon signed rank test and, to correct for multiple comparisons, a threshold of p<0.01 was used to denote significance. This reveals decrease in burden of participation coefficient nodes with a z-score of <-2 (p=0.000003)

| AGE AT EARLY SCAN | AGE AT LATE SCAN | SEX | POST OPERATIVE OUTCOME | HISTOPATHOLOGICAL FINDINGS | LATERALITY | LOBE |
| --- | --- | --- | --- | --- | --- | --- |
| 13.58 | 14.69 | 'M' | SF | 'FCD' | 'L' | 'Temporal' |
| 4.01 | 5.29 | 'F' | NSF | 'FCD' | 'R' | 'Frontal' |
| 2.26 | 2.89 | 'M' | SF | 'FCD' | 'L' | 'Temporal' |
| 11.1 | 15.22 | 'F' | SF | 'LEAT' | 'R' | 'Temporal' |
| 9.84 | 12.99 | 'M' | NSF | 'Other' | 'L' | 'Frontal' |
| 13.37 | 15.98 | 'F' | SF | 'LEAT' | 'L' | 'Temporal' |
| 15.39 | 15.58 | 'F' | SF | 'HS' | 'L' | 'Temporal' |
| 2.44 | 4.44 | 'M' | NSF | 'HS' | 'L' | 'Temporal' |
| 14.72 | 15.12 | 'F' | SF | 'LEAT' | 'L' | 'Temporal' |
| 8.22 | 9.77 | 'M' | SF | 'FCD' | 'L' | 'Frontal' |
| 8.85 | 9.93 | 'M' | NSF | 'FCD' | 'L' | 'Frontal' |
| 14.87 | 15.5 | 'F' | NSF | 'Other' | 'L' | 'Frontal' |
| 6.4 | 8.11 | 'M' | SF | 'Other' | 'R' | 'Temporal' |
| 7.32 | 8.72 | 'M' | SF | 'FCD' | 'R' | 'Frontal' |
| 14.85 | 17.24 | 'M' | NSF | 'Other' | 'L' | 'Frontal' |
| 16.05 | 18.17 | 'F' | SF | 'Other' | 'L' | 'Parietal' |
| 16.59 | 17.43 | 'F' | SF | 'Other' | 'L' | 'Temporal' |
| 16.1 | 16.24 | 'F' | SF | 'HS' | 'R' | 'Temporal' |
| 11.2 | 12.58 | 'F' | SF | 'HS' | 'L' | 'Temporal' |
| 1.78 | 2.54 | 'F' | SF | 'FCD' | 'R' | 'Frontal' |
| 7.93 | 9.08 | 'F' | NSF | 'FCD' | 'L' | 'Parietal' |
| 3.04 | 4.08 | 'M' | SF | 'LEAT' | 'R' | 'Occipital' |
| 11.32 | 12.24 | 'F' | SF | 'LEAT' | 'R' | 'Frontal' |

**Supplementary Table 1: Demographics of patients included in the study.**
